# Relational continuity of care in integrated maternity and child health clinics improve parents’ service experiences

**DOI:** 10.5334/ijic.1451

**Published:** 2014-10-29

**Authors:** Miia Tuominen, Anne Kaljonen, Pia Ahonen, Päivi Rautava

**Affiliations:** Department of Clinical Medicine, Public Health, University of Turku; Institute for Child and Youth Research, University of Turku, Turku, Finland; Institute for Child and Youth Research, University of Turku, Turku, Finland; Education and Research; Health and Well-being, Turku University of Applied Sciences, Turku, Finland; Department of Clinical Medicine, Public Health, University of Turku; Turku Clinical Research Centre, Turku University Hospital, Turku, Finland

**Keywords:** comparative study, child health services, maternal health services, continuity of patient care, STEPS study

## Abstract

**Introduction:**

In the Finnish primary health care, relational continuity of care is implemented in integrated maternity and child health clinics where the same nurse takes care of the family from the pregnancy until the child reaches school age. The aim of this study was to clarify the association between this relational continuity of care and the availability, utilisation and selected features of the maternity and child health clinic services, as evaluated by the parents.

**Methods:**

A comparative, cross-sectional service evaluation survey was used. Eighteen months after their baby's delivery, mothers (*N* = 987) and fathers (*N* = 835) from Southwest Finland evaluated specific maternity and child health clinic services. Comparisons were made between the parents who had relational continuity of care in the integrated maternity and child health clinics and those who did not.

**Results:**

Home visits were more frequently provided when relational continuity of care in integrated maternity and child health clinics existed. Parents who had this relational continuity of care, evaluated several features of the service, especially provided support, more positively than parents who did not.

**Conclusions:**

Relational continuity of care in integrated maternity and child health clinics seems to increase parents’ satisfaction with the services and might increase the provision of home visits.

## Introduction

Continuity of care has been shown to be related to patients’ increased satisfaction [1–3] and decreased utilisation of tertiary health care services [1, 4]. Continuity of care has also been recognised as one of the key elements of patient-centred care [5]. According to the review of Haggerty et al. [6] three types of continuity of care can exist in all health care settings: informational, management and relational continuity of care. Relational continuity of care appears in the ongoing relationship between the patient and her/his family and the care provider. In the field of maternity care, this has often been defined as the circumstances where the same midwife or small group of midwives takes care of the woman and family throughout pregnancy and childbirth, and into the early post-partum period [7, 8]. However, Haggerty et al. have proposed that continuity cannot exist without patients’ and their families’ real experience of coordinated, coherent and stable care which is based on their individual needs and context [6]. This underlines the importance of exploring factors that contribute to clients’ experiences of continuity in different health care settings.

This study focuses on relational continuity of care in the context of Finnish maternity and child health clinics. This means a long-term continuity of care, implemented by the same nurse from early pregnancy until the child reaches school age (the age of 7), which is built into the organisational model of the integrated maternity and child health clinic.

Finnish communal maternity and child health clinics are led by the registered nurses (public health nurses and midwives) with general practitioners. Other specialists, such as psychologists and social workers, are also involved in clinics’ free services [9, 10]. Practically, all families use the maternity and child health clinics; only 0.2%–0.3% of childbearing families are estimated to be non-users [11]. Parents in Finland are mainly satisfied with the services of maternity and child health clinics [12, 13] but also critical evaluations have been presented regarding specific aspects of the service [14–16].

The recent Government Decree (2011) regarding maternity and child health care and other preventive health services specifies that multiprofessionally implemented antenatal training should be arranged for first-time parents and at least one home visit must be made during the pregnancy or post-natal period [9, 17]. In addition, there are national non-obligatory recommendations that provide guidelines for the clinics’ operation [18–20]. Although provision of these services is dictated by the law, the organisational models of maternity and child health clinics are not. Thus, a great structural diversity exists [21, 22].

The aim of this study was to clarify the association between the relational continuity of care and the availability, utilisation and selected features of the maternity and child health clinic services, as evaluated by the parents. The research question was: Does relational continuity of care in integrated maternity and child health clinics improve parents’ service experiences?

Our underlying hypothesis was that the relational continuity of care would have a positive impact on the parents’ service experiences.

## Methods

### Design and sample

A cross-sectional service evaluation design was used. The study was part of the multidisciplinary STEPS study that is being carried out in the catchment area of the Turku University Hospital by the Institute for Child and Youth Research at the University of Turku. This prospective STEPS study is based on a cohort of all Finnish- or Swedish-speaking women who had live deliveries in the Hospital District of Southwest Finland from January 2008 to April 2010 (*n* = 9811) and their children (*n* = 9936). Women who were unable to communicate in Finnish or Swedish were excluded (*N* = 661). Out of this cohort population, 1797 women (18.3%) and their 1658 spouses were recruited to an intensive follow-up group in maternity health clinics during early pregnancy from September 2007 to August 2009, and at the hospital, during the intrapartum care from September 2007 to March 2010. These families will be followed up until the children are young adults. The parents gave a written informed consent, and they have been informed of their right to withdraw from the study at any point. The Ministry of Social Affairs and Health and the Ethics Committee of the Hospital District of Southwest Finland have approved the STEPS study. The STEPS study protocol has been previously reported in more detail [23].

The participating parents were asked questions regarding the maternity and child health clinic services as a part of the multidimensional questionnaires three times during the STEPS study follow-up: in early pregnancy, 4 months after delivery and 18 months after delivery. The present data were collected by a postal questionnaire 18 months after delivery. Approximately half of the STEPS study's participating mothers (*N* = 987, 54.9%) and fathers (*N* = 835, 50.4%) returned the questionnaire for this phase of the study. Information regarding parents’ background characteristics and family's socio-economic situation collected from mothers during early pregnancy and the 18 month follow-up was also used (Table 1).

**Figure 1.**
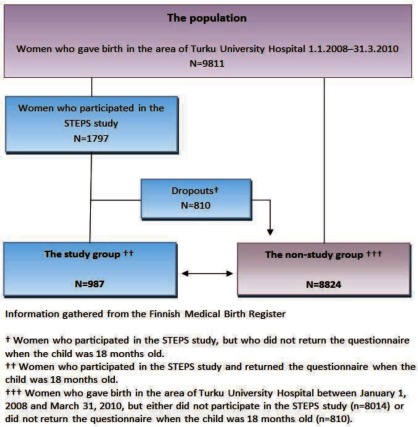
The formation of the study group and the drop-out analysis (women)

In the dropout analysis, the background characteristics of the participating mothers were compared with the data of mothers who had given birth in the area of the Turku University Hospital between 1 January 2008 and 31 March 2010. Their background data were obtained from the National Birth Register [24], which contains information on mothers and children (Figure 1). The characteristics of the participating and non-participating fathers could not be compared due to the lack of information on them in the National Birth Register.

### Measures

The questionnaire for parents included questions previously validated in the study by Viljamaa [12], which evaluated maternity and child health clinic services in Central Finland. Questions were selected and modified for this study by experts of the 10 Points project of the Turku University of Applied Sciences [25]. The questionnaire contained 75 questions, of which 22 were analysed and reported in this study.

The outcome measures of the study were the following:

1. Availability and utilisation of the maternity and child health clinic services.
2. Parents’ maternity and child health clinic service experiences.

**Figure 2.**
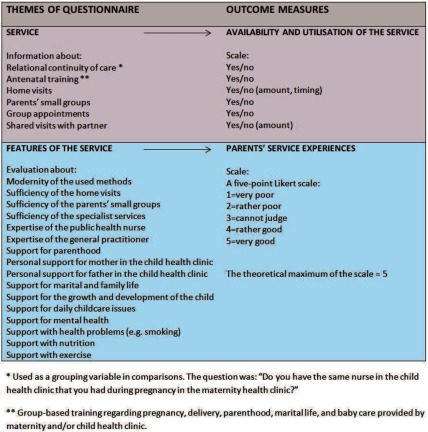
The composition of the questionnaire for the evaluation of the maternity and child health clinic services

The composition of the questionnaire and outcome measures are presented in Figure 2.

### Analytic strategy

The data were analysed statistically using SPSS 18.0 and SAS Release 9.1 for Windows. The reliability of the section of the questionnaire measured with a Likert scale, concerning parents’ service evaluations (16 variables), was estimated using Cronbach's alpha coefficient. The Cronbach's alpha for this section was 0.879 for mothers and 0.897 for fathers.

Descriptive statistics were calculated in terms of frequency, percent distribution, mean and standard deviation. The limit for statistical significance was set at *p* < 0.05. The relational continuity of care was set as an explanatory variable for the comparative analysis. The outcome variables regarding the evaluation of the maternity and child health clinic services were dichotomously classified as ‘good’ (very good + rather good) and ‘not good’ (very poor + rather poor + cannot judge). The value ‘good’ was set to indicate satisfaction with the service.

The Pearson's chi-squared test was first used to compare differences in the percentages of the groups (between the parents who had relational continuity of care in the maternity and child health clinics and those who did not). Binary logistic regression analysis was used to standardise the effect of significant confounding background factors (marital status, mothers and fathers age, professional education, level of income and parity) to outcome measures. The confidence interval was set at 95% in all analyses.

## Results

### Sociodemographic background of the participants

The essential sociodemographic variables of the participating women and of the non-participating parturients in the area of the Turku University Hospital are presented in Table 2. The participants were a little older, more often primiparous and married, and more of them than the non-participants were working as experts. In addition, the participants had undergone fewer abortions than the non-participants.

### Availability and utilisation of the maternity and child health clinic services

The availability and utilisation of the maternity and child health clinic services in relation to the relational continuity of care is presented in Table 3.

All respondent mothers had used the maternity and child health clinic services. The home visits were mainly provided after the delivery: the number of these post-natal home visits ranged from one to five. The majority of mothers who reported having had post-natal home visits had received one home visit (71.0%, *N* = 303), a quarter had received two visits (25.7%, *N* = 110) and a small proportion reported three or more (3.3%, *N* = 14) visits.

Small groups for parents and group appointments were infrequently provided and utilised in clinics. Mothers who had participated in some group activities reported small groups such as ‘mother and baby group’, ‘physiotherapy group’, ‘extended family training’, ‘family group’ and ‘mothers’ cafe’.

The availability of home visits, antenatal training and small groups for the parents was examined in relation to the parity of the women. A home visit had been provided for less than half of both the primiparous women (42.7%, *N* = 188) and the multiparous women (44.3%, *N* = 241). Antenatal training had been included in the maternity health clinic services for the majority of the primiparous women (91.6%, *N* = 404) and about half of the multiparous women (54.9%, *N* = 298). Small groups for parents were included in the maternity and child health clinic services for more than a quarter of primiparous women (30.2%, *N* = 130) and of less than a fifth of multiparous women (18.5%, *N* = 99). There was significant difference between the primiparous and multiparous women regarding the availability of antenatal training (*p* < 0.001) and small groups for parents (*p* < 0.001).

The information about the relational continuity of care in maternity and child health clinics was reported by 97.2% (*N* = 959) of the respondent mothers. The majority of these mothers (83.0%, *N* = 796) had a different nurse during pregnancy, in a separate maternity health clinic and after the delivery, in a separate child health clinic, thus relational continuity of care was not implemented for them. Relational continuity of care had been implemented in less than a fifth (17.0%, *N* = 163) of the cases; these mothers had the same nurse in integrated maternity and child health clinic.

Relational continuity of care was associated with the availability of home visits and with the manner of visiting: that is, the mother visited the clinics with her partner. In the binary logistic regression analysis primiparity (*p* < 0.001, OR 5.79, 3.63–9.21) and mothers’ (*p* < 0.001, OR 2.71, 1.78–4.13) and fathers’ younger age (*p =* 0.010, OR 1.86, 1.16–2.99) explained the continuity of care's impact on the frequency of visits, with a partner, to the clinics (Table 3).

### Parents’ evaluations regarding maternity and child health clinic services

Parents’ evaluations of received maternity and child health services ranked as ‘good’ are presented in the Table 4. The feature of the services most frequently ranked as ‘good’ by the mothers was ‘support for the growth and development of the child’, and by the fathers it was ‘expertise of the nurse’. The worst evaluation of the services by the mothers was for the feature ‘sufficiency of the parents’ small groups’, and by the fathers it was ‘support for mental health’.

The descriptive analysis showed that the proportion of the rankings marked ‘cannot judge’ regarding clinics’ services was generally high; this ranged between 3.7% and 86.3% for mothers, and 21.7% and 79.9% for fathers. Fathers were generally less able to evaluate services than mothers. The feature of the maternity and child health clinic services most frequently ranked as ‘cannot judge’ by the mothers was ‘support with health problems’ (86.3%, *N* = 843) and by the fathers it was ‘sufficiency of specialist services’ (79.9%, *N* = 651). The service feature least frequently ranked as ‘cannot judge’ by the mothers was ‘support for the growth and development of the child’ (3.7%, *N* = 66) and by the fathers it was ‘expertise of the nurse’ (21.7%, *N* = 178).

The sufficiency of home visits and specialist services; the received support for parenthood, exercise and health problems and personal support for the mother and for the father in the child health clinics were more often evaluated as ‘good’ by the mothers who had experienced relational continuity of care within the maternity and child health clinics. Furthermore, sufficiency of home visits and support with health problems and for mental health was evaluated as ‘good’ more often by fathers who had experienced relational continuity of care in these clinics (Table 4).

Most of the background variables did not explain the effect of relational continuity of care to outcome measures: only father's higher professional education (*p* = 0.020, OR 1.65, 1.08–2.52) explained the effect that relational continuity of care had on their good experiences with support for mental health given by the clinics. All the significant differences observed with service evaluations accumulated in favour of a relational continuity of care-based maternity and child health clinic services (Table 4).

## Discussion

Parents from Southwest Finland benefit from relational continuity of care in the integrated maternity and child health clinics. It seems to increase parents’ satisfaction with maternity and child health clinic services and supports the provision of home visits. The benefits of relational continuity of care enabled by integrated maternity and child health clinics appeared mainly through two dimensions of the service: home visits and support.

A relational continuity of care that exists in the integrated maternity and child health clinics was seen minimally in this study. Indeed, this model is rather uncommon nationally, as only 20% of Finnish municipalities provide maternity and child health clinic services as an integrated clinic [22]. Similar integration of maternity and child health services is very uncommon also globally and hardly any literature related to this particular clinic model exists. Debate about the best organisational model for the maternity and child health clinics has been going on for long in Finland. Experts have not agreed whether primary maternity and child health care should be provided by separated [26] or by integrated clinics [27], and lack of comparative evidence has made consistent development of these services challenging. Therefore, the strength of this study is that it gave more understanding about the meaning of organisational model and continuity of care in the context of maternity and child health clinics. In the field of integrated care one of the key concerns raised in global discussion is ‘What interventions should be packaged together?’ [28]. The association between parents’ positive service experiences and the relational continuity of care in integrated maternity and child health clinic found in this study may be interpreted as one answer to this question. Furthermore, the results of our present and former findings [16] may be used as the reasoning for more extensive integration of the maternity and child health clinic services in Finland.

However, integrated clinic is not the only method to promote continuity in maternity and child health care; it can be successfully supported in diverse organisational structures by implementing practices that foster relational coordination of work between the professionals [29] and enable concrete working together within shared strategy [30]. In the Nordic countries, integrated care that support continuity is been developed in special family centres, which gather together services for families to enable a smooth cross-sector collaboration of health and social care professionals, third sector and voluntary workers [31]. The current National Development Programme for Social Welfare and Health Care includes a statement related to expanding these family centres in Finland [32]. Reinforcement of this kind of coordination and integration of care would essentially promote continuity of care in family services.

Home visits, implemented by nurses, have been a recommended method in Finnish primary health care since the 1920s. However, although at the end of the 1980s, home visits were provided for almost all families with a newborn baby [33]; after the new millennium no more than two-thirds of nurses provided a home visit for every family with a newborn [21]. Our study confirms that home visits are still a rather limited service for families: only half of the mothers in this study reported one or more home visit, and the availability of these visits was often evaluated as insufficient. The scarcity of home visits provided by maternity and child health clinics has also been reported in a recent national study [13].

One main finding of our study was that relational continuity of care in maternity and child health clinics seems to increase the frequency of the provision of home visits. This is consistent with our previous results [16], and it also supports the conclusion that a model of integrated maternity and child health clinic may positively affect nurses’ readiness to implement home visits. Reinforcing implementing of integrated maternity and child health clinics might be one approach for achieving the now-required provision of home visits.

According to our results, it seems that the relational continuity of care has a positive impact on parents’ evaluations of several features of maternity and child health clinic services. This confirms the hypothesis generated from the previous studies [1–4, 6, 12, 16] that there could be an association between the relational continuity of care in maternity and child health clinics and parents’ positive service experiences. The differences between parents’ evaluations were clearly manifested in the experiences of the support provided by clinics. First, mothers evaluated the general support for parenthood and personal support for mother and for father in the child health clinic as better when a relational continuity of care in the maternity and child health clinic existed. Second, when this continuity existed, both parents evaluated the support for health problems more highly, and mothers evaluated support for exercise as better.

The support for parenthood is a key mission of the maternity and child health clinic services [9, 10, 17–21]. According to earlier studies, parents [13] and their public health nurses [34] have frequent worries regarding the health and psychosocial development of child as well as about parenthood. Further, dissatisfaction with the quality of their sexual relationship with their partner, [35] as well problems in the marital relationship are commonly reported by fathers with small children [36]. That means, parents with small children need professional support, especially for parental and marital issues, upbringing and child care [37, 38]; a health professional's familiar, supportive manner of working is thus highly appreciated by the parents [39]. Despite this, the impact of the relational continuity of care in maternity and child health clinics on the support provided, and on parents’ experiences of that support, is still largely unknown. Irrespective of the lack of proper comparable research, there is indicative evidence regarding relational continuity of care and parents’ positive perceptions of being supported with breastfeeding in maternity and child health care [40]. Furthermore, it has been suggested that the model of an integrated maternity and child health clinic, which enables relational continuity of care, may improve family-centred health promoting counselling [41] and would create a propitious basis for confidential cooperation between families and nurses when mental health problems exist [42].

Therefore, we suggest that parents’ experiences of being well supported by the professionals at maternity and child health clinics might be associated with the relational continuity of their care. However, the lack of comprehensive evidence regarding a relational continuity of care within integrated maternity and child health clinics mean that further research is necessary.

This study does have some limitations. First, the proportion of the ‘cannot judge’ rankings was surprisingly high, especially in fathers. This might be a sign of poor awareness in fathers of the variety of the maternity and child health clinic services, despite couples frequently visited the clinics together. Supporting this idea, there is previous evidence revealing that fathers can feel themselves to be bystanders in the maternity and child health clinics [43, 44] which might complicate a father's capability to objectively evaluate the services. However, in Finland, support for fatherhood and equality between the parents in health services are required by national guidelines [9, 17–20]. Therefore, the parents’ common inability to evaluate their maternity and child health clinic's services raises the question of how the goals and procedures of the clinics were introduced to them by the personnel. Another explanation for the high proportion of ‘cannot judge’ rankings is the disparity between the maternity and child health clinics in the content of their services and organisational structure which meant that some of the evaluated services were not equally provided in all clinics. This made an inclusive evaluation problematic for the parents.

Second, the participation rate for the STEPS study was low (18.3%). This might diminish the generalisability of the prevalence estimates; however, the associations between the explored variables could be interpreted without bias [45]. The reasons behind the low participation rate of the STEPS study have been discussed elsewhere in detail [23]. A comparison of the background characteristics of the women in our study and a similar cohort from the National Birth Register suggests that the study group satisfactorily describes the non-study group in relation to the obstetric background variables, although some differences were observed. In the light of our research question, the clinical importance of these observed significant differences between the groups could be considered as minor. This was also supported by the logistic analyses. The similarity between participating and non-participating fathers could not be defined due to a lack of comparable background characteristics for them.

Parents’ satisfaction is crucial for high-quality maternity and child health services, but is in itself not enough. The development of maternity and child health services should always be based on rigorous evaluation of the health outcomes for the mother and the baby. Therefore, our future research will focus on the assessment of maternal and perinatal outcomes in relation to the organisational model of maternity and child health clinics.

## Conclusion

Our results suggest that the relational continuity of care that exists in the organisational model of an integrated maternity and child health clinic may increase parents’ satisfaction with the specific features of the service and support a greater provision of home visits. Relational continuity of care in primary maternity and child health care seems to benefit parents.

## Figures and Tables

**Table 1.**
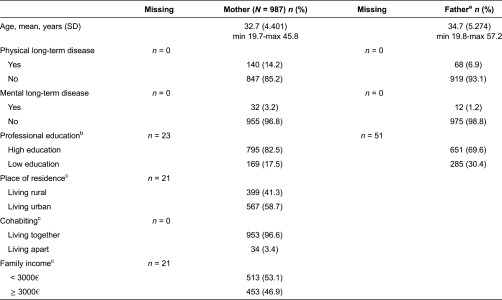
Sociodemographic characteristics of the participating parents in the STEPS study's 18 month follow-up

^a^Information regarding fathers is based on the report of the mothers (*N* = 987).

^b^High education = degree from university, polytechnic/university of applied sciences or college, low education = other degrees/no degrees.

^c^Information regarding family's situation is based on the report of the mothers.

**Table 2.**
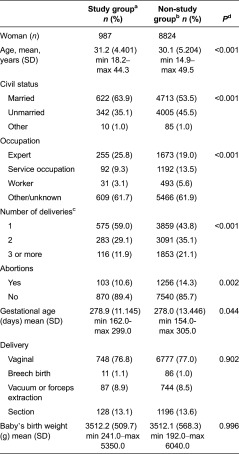
National Birth Register characteristics of the participant mothers – a comparison between participants and non-participants

Information gathered from the Finnish Medical Birth Register (2013).

^a^Women who participated into the STEPS study's 18 months follow-up and gave birth between 1 January 2008 and 31 March 2010 in the area of the Turku University Hospital.

^b^Women who did not participate into the STEPS study and gave birth between 1 January 2008 and 31 March 2010 in the area of the Turku University Hospital.

^c^Defined at the STEPS study's 18 months follow-up.

^d^Used statistical test: Pearson's chi-square.

**Table 3.**
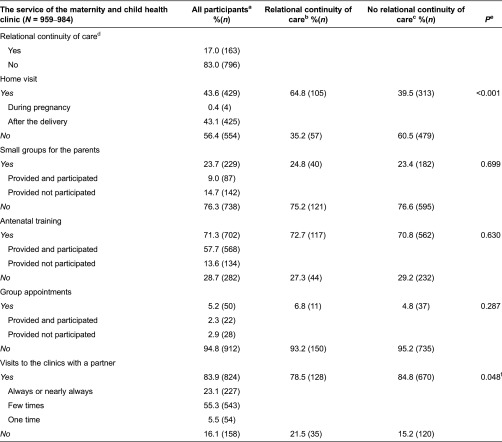
The availability and utilisation of the maternity and child health clinic services in relation to the relational continuity of care reported by the mothers (*N* = 987)

^a^Includes participants without information on relational continuity of care.

^b^The same nurse takes care of family in the maternity health clinic and child health clinic.

^c^Different nurses take care of the family in the maternity health clinic and child health clinic.

^d^Information on relational continuity of care was known of 97.2% (*N* = 959) of the study participants. Because of this and the different response rates between the questions, a variation in total and group-based frequencies and percentages exists.

^e^The statistical test (Pearson's chi-square) was performed for the dichotomised variables (yes/no).

^f^Difference was explained by the primiparity and parents’ lower age in the binary logistic regression analysis.

**Table 4.**
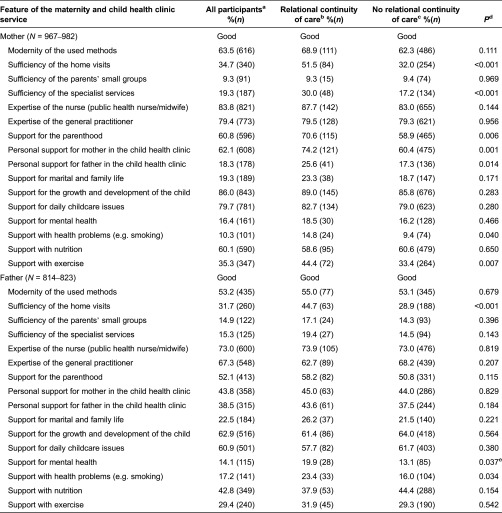
Mothers’ (*N* = 987) and fathers’ (*N* = 835) ‘good’ service evaluations in relation to relational continuity of care in the maternity and child health clinics

Information of relational continuity of care was known of 97.2% (*N* = 959) of the study participants. Because of this, and the different response rates between the questions, a variation in total and group-based frequencies and percentages exists.

^a^Includes participants without information on relational continuity of care.

^b^Same nurse takes care of family in maternity health clinic and in child health clinic.

^c^Different nurses take care of family in maternity health clinic and in child health clinic.

^d^Used statistical test: Pearson's chi-square.

^e^Difference was explained by partners’ higher professional education in the binary logistic regression analysis.
